# Prevalence of Veterinary Antibiotics and Antibiotic-Resistant *Escherichia coli* in the Surface Water of a Livestock Production Region in Northern China

**DOI:** 10.1371/journal.pone.0111026

**Published:** 2014-11-05

**Authors:** Xuelian Zhang, Yanxia Li, Bei Liu, Jing Wang, Chenghong Feng, Min Gao, Lina Wang

**Affiliations:** State Key Laboratory of Water Environment Simulation, School of Environment, Beijing Normal University, Beijing, China; Kent State University, United States of America

## Abstract

This study investigated the occurrence of 12 veterinary antibiotics (VAs) and the susceptibility of *Escherichia coli* (*E. coli*) in a rural water system that was affected by livestock production in northern China. Each of the surveyed sites was determined with at least eight antibiotics with maximum concentration of up to 450 ng L^−1^. The use of VAs in livestock farming probably was a primary source of antibiotics in the rivers. Increasing total antibiotics were measured from up- to mid- and downstream in the two tributaries. Eighty-eight percent of the 218 *E. coli* isolates that were derived from the study area exhibited, in total, 48 resistance profiles against the eight examined drugs. Significant correlations were found among the resistance rates of sulfamethoxazole-trimethoprim, chloromycetin and ampicillin as well as between tetracycline and chlortetracycline, suggesting a possible cross-selection for resistance among these drugs. The *E. coli* resistance frequency also increased from up- to midstream in the three rivers. *E. coli* isolates from different water systems showed varying drug numbers of resistance. No clear relationship was observed in the antibiotic resistance frequency with corresponding antibiotic concentration, indicating that the antibiotic resistance for *E. coli* in the aquatic environment might be affected by factors besides antibiotics. High numbers of resistant *E. coli* were also isolated from the conserved reservoir. These results suggest that rural surface water may become a large pool of VAs and resistant bacteria. This study contributes to current information on VAs and resistant bacteria contamination in aquatic environments particularly in areas under intensive agriculture. Moreover, this study indicates an urgent need to monitor the use of VAs in animal production, and to control the release of animal-originated antibiotics into the environment.

## Introduction

Antibiotic and antibiotic-resistant microbial contamination is an issue of growing global concern, both in the public and in the research community. The rampant use of antibiotics in medicine and agriculture has resulted in the extensive detection of antibiotics, antibiotic-resistant bacteria and antibiotic resistance genes (ARGs) in the environment worldwide, including in 139 streams in the US, the Osaka area of Japan, the Haihe River and the Yangtze estuary in China [1]–[4].

Animals in concentrated feeding operations are the chief consumers of antimicrobial agents, which are administered for growth improvement and disease control. The amount of veterinary antibiotics (VAs) annually administered in the world reaches 10^5^–10^6^ tonnes [5]–[7]. Guidelines have existed that ban the use of certain antibiotics as growth promoters in both the European Union and the US since 1999, but this prohibition has led to a corresponding increased use of VAs for disease control and improving feed efficiency [8]. Considering Denmark as an example, the total veterinary use remained as high as 107 t in 2011 [9], [10]. Antibiotics are poorly absorbed in the animal gut; as a result, approximately 40–90% of these antibiotics will be excreted as parent compounds or metabolites via urine or feces [11], [12], and the residue of VAs in animal wastes has been widely reported [7], [13]–[16].

Animal wastes are usually stored using a lagoon system and/or are composted instead of intensive treatment before being discharged from animal farms [10]. However, both of these methods are limited in their ability to completely remove antibiotics. For instance, compost can reduce antibiotics by 20%–99% [17], [18], and a lagoon system can decrease tylosin by no more than 75% [19]. Thus, high amounts of antibiotics might remain in animal waste and are a potential pollution source of environmental antibiotics. Residual antibiotics in post-treated animal wastes can be disseminated into the surrounding aquatic environment via runoff when utilized as fertilizer on farmlands [20], [21] or in some cases, when directly released to receiving watersheds through sewage discharge or occasional leaching from animal farms. Such scattered pollution sources from operations as well as from fertilized fields will lead to more severe and complex antibiotic contamination in these rural areas compared to point-source-affected urban rivers; therefore, the antibiotic contamination in these regions requires additional attention and study.

The particular concern over VAs exposure is the increased presence of antibiotic resistant bacteria in the environment. Antibiotic-resistant bacteria might develop resistance within animal bodies from exposure to administered VAs [22], which can be excreted in feces and be subsequently released into the environment along with antibiotics [23], [24]. Furthermore, long-term exposure to sub-therapeutic levels of antimicrobial agents in aquatic environments imposes selection stress on environmental microorganisms [25]. Once antibiotic-resistant microorganisms appear in the environment, they enlarge the resistance community through the transfer of corresponding ARGs among microbial populations [3], [26]. In addition, antibiotic-resistant bacteria are likely to transport vertically and horizontally through physical or biological media [27]. Since livestock breeding and related agricultural activity is a potential source of antibiotics and resistant bacteria in the environmental, there is an urgent need to elucidate how antibiotics, and particularly antibiotic-resistant fecal indicator bacteria (*E. coli*) contamination in rural aquatic environments, might be impacted by intensive concentrated animal feeding operations (CAFOs).

China is the largest animal feeding country in the world and is experiencing the expansion and intensification of animal feeding operations in many areas. Due to the absence until now of relevant regulation, the residues of a variety of VAs have been consistently reported at high levels in animal waste [7], [13], [15]. Nevertheless, limited information is available providing baseline data about surface water pollution with antibiotics and antibiotic-resistant bacteria in typical livestock production regions. Therefore, this study investigated the Jiyun River, Beijing, which flows through Pinggu County, which is one of the primary meat-providing counties in Beijing, to (1) determine the occurrence and spatial distribution of antibiotics in the water system, (2) examine the antibiotic susceptibility of *E. coli* in this basin and (3) explore the correlation between *E. coli* resistance and antibiotic concentrations. We seek to provide useful information of the influence that is exerted on the aquatic ecosystem by VAs administration in the animal industry.

## Materials and Methods

### Study area, sampling sites and sample collection

The study area of Pinggu County is located east of Beijing, China. This county has the highest animal feeding density among the suburbs of Beijing and provided 3.4×10^5^ and 7.11×10^6^ swine and poultry products, respectively, to capital market in 2011 [28]. The main stream of the Jiyun River is Ju River, with a stretch of 54.4 km, which flows across Pinggu County over an area of 1352 km^2^ and eventually enters Bohai Bay.

**Figure 1:**
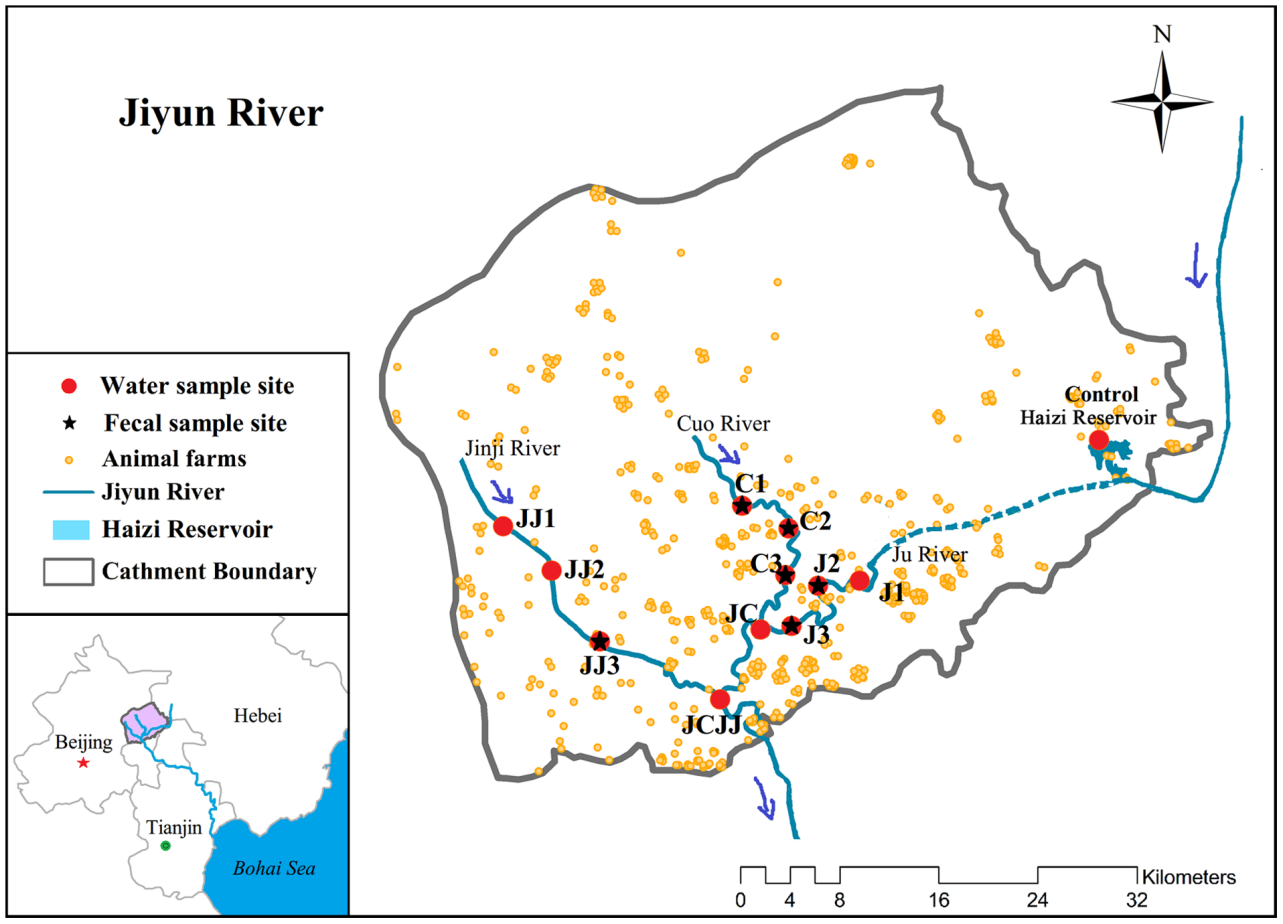
Map of sampling sites in the Jiyun River.

The Jiyun River has two tributaries: Cuo River and Jinji River. The sampling sites were selected along the upper, middle and lower reaches of the main stream and two tributaries. Eight of the 9 sites were directly adjacent to animal farms. These animal farms are small-scale operations, and none of the 8 farms was equipped with a professional lagoon system or fecal treatment procedure. The effluents of these farms are directly discharged into adjacent water bodies. The fecal wastes were simply piled up with maize straw in the air, but usually without any bulking agents, and then applied to nearby farmlands during fertilizer season. Six fresh fecal samples were collected from six of the eight animal operations with the permission of farmers, but samples from the other two farms were not available. From each farm, multiple points of fresh feces were obtained from the pigpen and were mixed to obtain a representative sample. Two intersection sites (JC and JCJJ) in the main stream Ju River separately receiving flow from Cuo River and Jinji River, respectively, and a site from the Haizi reservoir were also selected. The GPS coordinates of each location in this study are listed in Table S1. The sampling locations and sampling activity in this study did not require specific permissions and the sampling did not involve endangered or protected species. The study area and sample sites distributions are presented in Figure 1. The sampling was conducted on May 16, 2013, and a total of 12 water samples and 6 fresh fecal samples were obtained.

The samples were separated into two parts: one part received sodium azide immediately to inhibit microbial activity and prevent antibiotic biodegradation for further antibiotic analysis [29], [30], and the other part was free of sodium azide for microbiological property determination and pure *E. coli* clone isolation. The samples were preserved in cold boxes, transported to the laboratory within one day and maintained at 4°C until use. The microbial samples were incubated and the pH values were measured immediately upon return to the laboratory. The antibiotics were detected beginning on the second day.

### Sample preparation and analysis

Twelve target antibiotics in three different groups (tetracyclines (TCs): tetraycline (TC), oxytetracycline (OTC), chlortetracycline (CTC) and doxycycline (DOC); quinolones (QLs): ciprofloxacin (CFC), enrofloxacin (EFC) and ofloxacin (OFC); and sulfonamides (SAs): sulfadiazine (SDZ), sulfamethoxazole (SMX), sulfamonomethoxine (SMM), sulfameter (SM), sulfachinoxaline (SCX)) were analyzed in this study.

The samples were pretreated according to the method of [12], [15] with slight modification. Briefly, a 2-L water sample was filtered through 0.45-µm pore glass fiber membrane filter. The filtered water sample was acidified to a pH of 3.0 with 30% H_2_SO_4_ (V/V), followed by the addition of Na_2_-EDTA (0.5 g L^−1^) as a chelating agent. The acidified water samples were concentrated by solid-phase extraction (SPE) over an Oasis HLB cartridge (6cc, 500 mg, Waters, USA) at a flow rate of 5 ml min^−1^. The antibiotics that were retained on the HLB cartridges were eluted with 10 ml of methanol (0.1% formic acid) after being dried with nitrogen gas at a flow rate of 5 ml min^−1^. The eluent was concentrated to near dryness under a gentle nitrogen stream in a 37°C water bath. A mixture of internal standard containing tetracycline-D6, enrofloxacin-D5 and sulfamethazine-D4 was added to compensate for the variations of TCs, FQs and SAs, respectively, during detection. Methanol was supplemented to a final volume of 1 ml. The final solution was filtered through a PTFE filter (Millipore) for liquid chromatography - tandem mass spectrometry (LC-MS-MS) analysis. For the solid samples, 3 g of fresh fecal samples were extracted with a 20-ml mixture of methanol and EDTA-McIlvaine buffer (V:V = 1∶1) with 30 minutes of rotation and ultrasonication, respectively, three times. The extracts were centrifuged and the supernatant was collected and diluted with deionized water to maintain a methanol proportion below 10%. The substantial purification and concentration followed the steps of the water samples.

The extracted samples were analyzed by LC-MS-MS. The separation of the target compounds was performed on a C18 column (250×4.6 mm, 5 mm; Akzo Nobel, Sweden) in a HPLC system (DIONEX UltiMate 3000). A binary elution gradient consisting of A (0.1% formic acid water solution) and B (methanol) was used in the following program: 0–2 min: 15–30% B, 2–5 min: 30–40% B, 5–15 min: 40–70% B, 15–17 min: 70–100% B, and 17–21 min 100% B. A triple quadrupole mass spectrometer (API 3200, AB-SCIEX, Framingham, MA) that was equipped with an electrospray ionization (ESI) source in positive ion mode was used. The multiple reaction monitoring (MRM) mode was used for the quantitation. The most intensive ion pairs together with retention time were used to identify the targeted antibiotics. Multipoint internal calibration curves were used to quantify the antibiotics. The recoveries of the 12 monitored compounds were in the range of 71 to 100%.

### *E. coli* isolation and confirmation

*E. coli* in the water was detected using the enzyme substrate method [31]. Briefly, Colilert substrates (IDEXX Laboratories, Inc., Westbrook, ME) were added to 100 ml water samples and were mixed thoroughly until all of the substrate dissolved per the manufacturer’s instructions. The dissolved water was sealed in an IDEXX Quanti-Tray/2000 (IDEXX Laboratories, Inc., Westbrook, ME) and incubated at 37°C for 24 h. The wells that changed to yellow and produced additional fluorescence were identified as harboring *E. coli*.

The *E. coli* isolates were obtained and confirmed according to previous descriptions [26], [31] with some minor revision. Briefly, 10 µl of a 10^−4^–10^−5^ dilution of liquid from 10–15 wells that were positive for *E. coli* growth on the plate for each site was streaked onto a Mueller-Hinton (MH) agar plate for incubation of 18 h at 37°C to obtain 10–50 clones on each plate. Approximately 4 isolates were randomly selected from well-separated colonies on each plate and purified on Mueller-Hinton (MH) agar plates using the crossed dilution method. To confirm these isolates, they were separately inoculated in *Escherichia coli* broth (EC broth) containing 4-methylumbelliferyl-, BD-glucuronide (EC-MUG) and tryptophan separately. The isolates that generated a blue fluorescence in EC-MUG and produced indole (identified by a rose-colored product after the addition of Kovacs agent) after 24 h of incubation at 37°C were confirmed as *E. coli*. In total, 218 isolates were collected from the study area. The confirmed isolates were re-streaked onto MH agar plates for subsequent susceptibility tests.

### Antibiotics susceptibility test

In total, eight drugs were tested for *E. coli* susceptibility primarily based on their detection in this study and their clinical importance. CTC and SMM tests were performed via growth measurement in Mueller-Hinton Broth in the presence and absence of antibiotics. The other six antibiotics were analyzed via the disk diffusion method [32]. The CTC and SMM test concentration was set to 25 µg and 500 µg per ml MH broth, respectively, according to a combination of prescribed doses in CLSI guidelines and the levels that have been used in other studies [33]–[35]. The CTC stock was directly prepared in MH broth. The SMM was dissolved in MH broth with a few drops of NaOH solution. A pure *E. coli* culture was suspended in sterile sodium chloride solution to 0.5 McFarland turbidity level after the incubation for 18 hours at 37°C. Fifty µl of a 10^−2^ suspension was used to separately inoculate wells containing 50 µl of MH broth with or without target antibiotic. The preliminary test indicated that the amount of NaOH remaining in SMM wells had no effect on *E. coli* growth. Therefore, the first and second wells in each group of the 4 wells were always the negative control (MH broth) and the positive control (MH broth with *E. coli* inoculum), respectively. After incubation for 22 hours at 37°C, the 96-well plate was read spectrophotometrically at 600 nm. The isolates were recorded as resistant to a particular antibiotic if the growth, as measured through OD values, was inhibited by less than 15% compared to that of the positive control or were recorded as susceptible when the growth was reduced by at least half [27], [33], [36].

The six antibiotic disks (Oxoid, UK) included ampicillin (AMP, 10 µg), chloromycetin (C, 30 µg), levofloxacin (LEV, 5 µg), tetracycline (TE, 30 µg), gentamycin (CN, 10 µg) and sulfamethoxazole-trimethoprim (SXT, 25 µg/1.25 µg). A 0.5 McFarland turbidity level of the *E. coli* isolate suspension was obtained using sterile sodium chloride solution after the isolates were incubated on MH agar plates at 37°C. The suspension was evenly inoculated onto an MH agar plate using autoclaved gauze. Three different disks were placed onto each inoculated plate. The disks were far away from each other and from the plate rim to avoid overlapping inhibition zones. The inhibition zone diameter was precisely measured using a ruler after 16–18 h of incubation at 37°C. The diameter was compared to the diameter of the susceptible, moderate and resistant standards as listed in CLSI 2009. The antibiotic resistance frequency was calculated as the ratio of *E. coli* isolates that were resistant to antibiotics to the total number that were isolated from each site. The multi-antibiotic resistance (MAR) index was estimated by the equation a/(b×c), where a is the total antibiotic resistance score of all of the isolates from the sample, b is the number of tested drugs, and c is the number of isolates from the sample [26]. The significance of difference in average *E. coli* resistance frequencies and MAR among the three rivers was examined through a non-parametric k independent samples Kruskal-Wallis test using SPSS 17.0. The correlations between *E. coli* counts and resistance frequencies or MAR as well as among the percent resistances to the eight drugs in the seven sites were examined via a Spearman correlation analysis using SPSS 17.0. The two sites, including the control and C1, were removed during the correlation analysis because of low antibiotic levels and few *E. coli* isolates, respectively.

## Results and Discussion

### Occurrences and levels of antibiotics in the Jiyun River

The detection frequencies and concentration profiles of the 12 monitored antibiotics are summarized in Table 1. The selected VAs were extensively detected in the investigated water system at percentages ranging from 58% to 100%. The concentrations varied largely among these sites from less than 1 ng L^−1^ to 450 ng L^−1^. Based on our determination, the SAs (SM, SMX and SDZ) and QLs (OFC, EFC and CFC) might be the predominant antibiotics in the water, as they were present in all of these investigated sites at an average concentration range of 3.79 to 92.97 ng L^−1^. In comparison, the TCs were generally one or two orders of magnitude lower (mean 2.17–16.12 ng L^−1^) than were the SAs (except for SCX) in concentration.

**Table 1:** Frequencies and concentrations of the 12 target antibiotics in the Jiyun River (n = 12).

Class	Compound	Frequency(%)(%)	Range (Mean)(ng L^−1^)(ng/L)	MDLs((ng L^−1^))
Tetracyclines (TCs)	Tetraycline (TC)	83.33	n.d-11.00 (2.17)	2.14
	Oxytetracycline (OTC)	91.67	n.d-100.00 (16.12)	2.35
	Chlortetracycline (CTC)	83.33	n.d-40.60 (12.92)	2.87
	Doxycycline (DOC)	58.33	n.d-11.75 (2.84)	2.45
Quinolones (QLs)	Ciprofloxacin (CFC)	100.00	3.56–24.80 (11.61)	2.15
	Enrofloxacin (EFC)	100.00	0.55–13.41 (3.79)	0.25
	Ofloxacin (OFC)	100.00	1.34–102.00 (27.89)	1.10
Sulfonamides (SAs)	Sulfadiazine (SDZ)	100.00	0.03–385.70 (62.45)	0.01
	Sulfamethoxazole (SMX)	100.00	4.29–230.00 (54.65)	1.15
	Sulfamonomethoxin (SMM)	75.00	n.d-450.00 (147.64)	1.10
	Sulfameter (SM)	100.00	0.51–387.00 (92.97)	0.16
	Sulfachinoxalin (SCX)	91.67	n.d-13.95 (2.90)	0.57

n.d: non-detected.

MDLs: method detection limitations for the 12 compounds.

**Figure 2:**
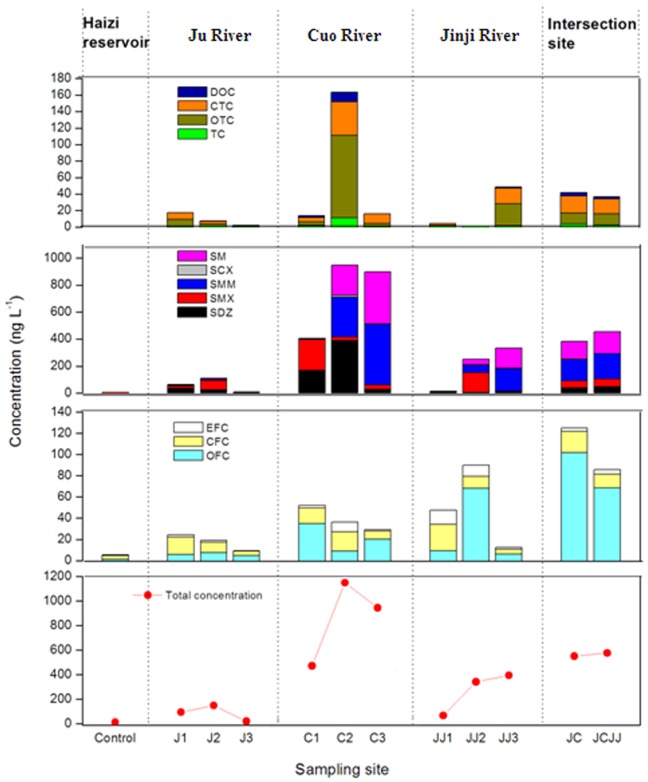
Distributions of the three classes of antibiotics in the 12 sampling sites. Control, Haizi reservoir; J1, J2 and J3, up-, mid- and downstream of Ju River; C1 C2, and C3, up-, mid- and downstream of Cuo River; JJ1, JJ2, and JJ3, up-, mid- and downstream of Jinji River; JC and JCJJ, intersection sites of Ju River with Cuo River and Jinji River, respectively. TC, tetracycline; OTC, oxytetracycline; CTC, chlortetracycline; DOC, doxycycline; CFC, ciprofloxacin; EFC, enrofloxacin; OFC, ofloxacin; SDZ, sulfadiazine; SMX, sulfamethoxazole; SMM, sulfamonomethoxine; SM, sulfameter; SCX, sulfachinoxaline.

Figure 2 depicts the antibiotic distributions in the Jiyun River. The three rivers displayed varying antibiotic abundances with approximately 4- and 2-fold greater average antibiotic levels at the 3 sites in the Cuo River and Jinji River tributaries than that in the main stream Ju River. The total antibiotic contents at mid- and downstream (JJ2 and JJ3, and C2 and C3) in the two tributaries increased compared to the upstream site (JJ1 and C1). Our results contrast with those of other studies investigating antibiotic variation in point-source-affected water bodies, in which decreased antibiotic concentrations were detected in the water over distances from the site receiving a point-source effluent due to natural attenuation by adsorption, dilution, photolysis, hydrolysis and biodegradation [37], [38].

The monitored sites, with the exception of Haizi reservoir, J1, JC and JCJJ, were immediately close to swine production farms, from which the sewage was directly discharged into receiving rivers, and the animal feces were used as a fertilizer in the nearby farmlands. Residual antibiotics in manure can concentrate, migrate in agricultural soil and finally end up in the aquatic environment through runoff when the feces are applied as fertilizer [20], [21]. Therefore, the potential continuous input of antibiotics from the discharge of animal farms as well as from the runoff of fertilized farmland was inferred to account for the accumulated antibiotic contamination along the river. In addition, high amounts of antibiotics were also recorded at the two sites where the Ju River intersected with the two tributaries: JC (Ju River/Cuo River) and JCJJ (Ju River/Cuo River/Jinji River) (Figure 2). The antibiotic concentrations at the three sites J1, J2 and J3 were almost the lowest of the surveyed sites in the three rivers, except for JJ1. One possible reason is due to the dilution and rapid transfer of antibiotics in large water flow of the main stream. However, the specific reason requires further study. High concentrations of antibiotics at the two intersection sites likely indicate potential compound transfer with flow from the tributaries into the main stream.

As seen in Figure 2, most of the surveyed sites in the Jiyun River were generally determined to have higher levels of SAs (SM, SMM, SMX and SDZ) and QLs (OFC and CFC) than TCs. These findings agree with the results of many other studies [4], [39], [40] and may be chiefly explained by the discrepant partition characteristics among different antibiotic classes. The TCs have strong combination abilities with soil/sediment and their mineral or organic components via cation bridging and/or cation exchange [41], [42], which causes their retention in the soil or dispersion to the sediment after being discharged into rivers. This result is supported by detection of TCs in the sediment of this river (Table S3). In contrast, most SA compounds predominantly exist in anionic species with negative charges at environmental pH values of >7 [43], [44] and are consequently less frequently adsorbed onto solid-phase material. The pH values of these water samples were in the range of 7.29 to 9.24. This range facilitates the dissolution and migration of SAs. Thus, it is reasonable that the SAs were primarily observed in the river water.

**Figure 3:**
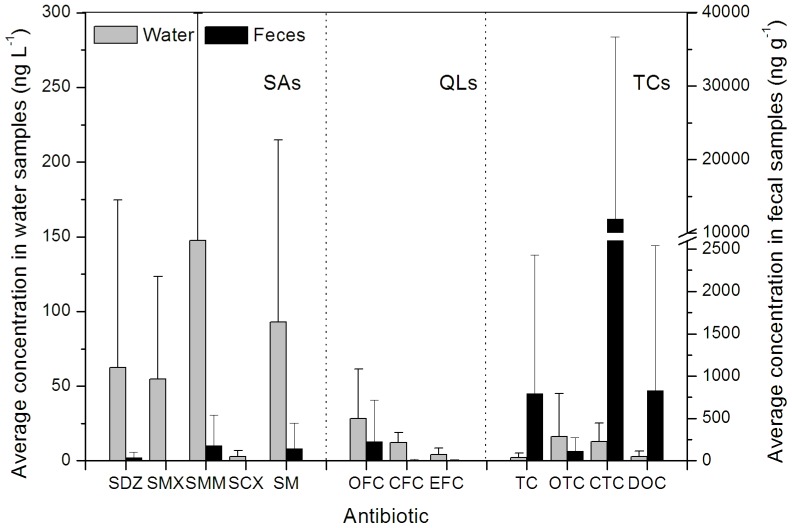
The means and standard deviations (SD) for antibiotics in water samples (n = 12) and in animal fecal samples (n = 6). TCs, tetracyclines; QLs, quinolones; SAs, sulfonamides; TC, tetracycline; OTC, oxytetracycline; CTC, chlortetracycline; DOC, doxycycline; CFC, ciprofloxacin; EFC, enrofloxacin; OFC, ofloxacin; SDZ, sulfadiazine; SMX, sulfamethoxazole; SMM, sulfamonomethoxine; SM, sulfameter; SCX, sulfachinoxaline.

A parallel survey of the antibiotic residues in six fecal samples of the animal operations that were directly adjacent to the water sample sites was conducted to explore the association of surface water antibiotic contamination with livestock production. The 12 target antibiotics were widely detected in these feces at levels of 1.03–56200 ng g^−1^. The water and fecal samples from the study area were generally coincident in the antibiotic composition of the two classes of VAs: SAs and QLs (Figure 3). Both of the matrixes were dominated by SM and SMM, and SCX was seldom detected. In addition, these matrixes presented the same order of QLs: OFC>CFC>EFC. In contrast, although large amounts of TCs were detected in these animal feces, they were found at relatively low levels in the water. This finding may be explained by the strong adsorption of TCs in the environment as discussed above and is also supported by the detection of TCs in the river sediment (Table S3).

The detection concentrations of VAs in the current study are lower than or comparable to the levels in other surface waters that are proximate to animal operations in Jiangsu, China (560–2420 ng L^−1^) [45] and in the US (1000–1500 ng L^−1^) [46]. These reported results and the concentrations in this study were significantly higher than in rivers receiving effluent from WWTPs, such as the Victoria River, HongKong, China (<20 ng L^−1^); the Elbe River, Germany (30–70 ng L^−1^); and the Ebro River, Spain (0.2–35.6 ng L^−1^) [47]–[49]. These results indicate that the influences of the rural aquatic ecosystem caused by livestock production are much greater than are those in urban areas caused by human populations.

### Antibiotic-resistance for *E. coli* clones that were isolated from the Jiyun River

**Figure 4:**
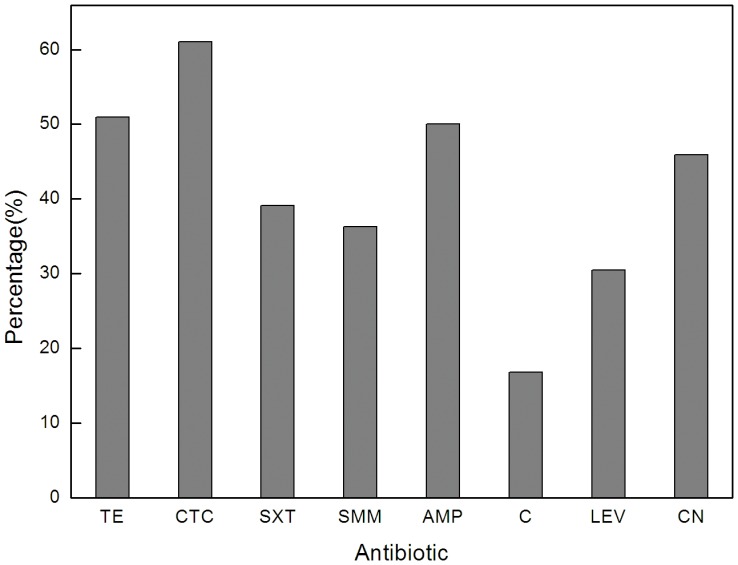
Resistance percentage to the eight tested drugs for all the *E. coli* isolates that were isolated from the Jiyun River (n = 218). TE, tetracycline; CTC, chlortetracycline; SXT, sulfamethoxazole-trimethoprim; SMM, sulfamonomethoxine; AMP, ampicillin; C, chloromycetin; LEV, levofloxacin; CN, gentamycin.

The detected *E. coli* counts in the Jiyun River were shown in Table S2. A total of 218 *E. coli* clones were isolated from the Jiyun River that not only exhibited a high percentage of resistance to the drugs that are frequently detected in these rivers but also to those that were not monitored but are of clinical concern. In total, 88% of the 218 isolates exhibited resistance to one or more of the tested drugs. The most frequent resistance appeared for CTC (61.01%), followed by AMP and TE (approximately 50%), CN (45.91%), SXT and SMM (39.09% and 36.24%), LEV (30.45%) and C (16.82%) (Figure 4). High levels of *E. coli* resistance to CTC, TE and AMP have been found in other aquatic environments [27], [33] and in WWTP [50]. The great resistance frequency against AMP in the environment was possibly because of its comparatively older utilization history (over years) [51]. This resistance also reflects the common use of AMP in agricultural activities [52]. The high resistance rate to CTC maybe is due to the potential long-term exposure of *E. coli* to CTC in these aquatic environments. The frequent TE resistance despite its low concentration in water column may be caused by the possibility that the majority of TE-resistant *E. coli* have developed resistance against TE before they enter rivers because high levels of TCs were identified in the surveyed animal feces. In addition, the tet genes (e.g., tet(A), tet(C) and tet(G)) readily spreading among gram-negative bacteria such as *E. coli* through transposons and smaller plasmids might also facilitate the abundance of TE resistant *E. coli* [39]. The relatively lower resistance frequency against C agrees with the observations of an earlier previous study [53].

**Figure 5:**
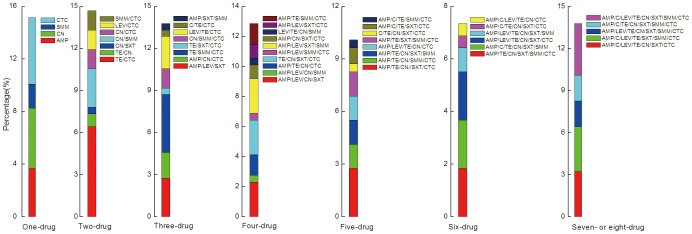
Percentage of *E. coli* number in each antibiotic-resistance pattern accounting for the total *E. coli* isolates from the Jiyun River. TE, tetracycline; CTC, chlortetracycline; SXT, sulfamethoxazole-trimethoprim; SMM, sulfamonomethoxine; AMP, ampicillin; C, chloromycetin; LEV, levofloxacin; CN, gentamycin.

Approximately 72% of these bacteria exhibited multiple antibiotic resistances, and, in total, 48 resistance profiles were determined (Figure 5). The five primary profiles were AMP, CN, CTC, TE/CTC and TE/SMM/CTC. The total rate of resistance to the five profiles for *E. coli* that were isolated from the Jiyun River was approximately 24%. It is worth noting that 3.67% of the isolates from the 9 sites exhibited eight-drug resistance. Ongoing multiple antibiotic exposure must have occurred in the study area as it has been repeatedly demonstrated that bacterial antibiotic resistance diversity is closely related to antibiotic contamination frequency [50].

Table 2 lists the relationships of the percent resistances among the eight drugs. Interestingly, significant correlations were obtained among the three drugs SXT, C, and AMP (p<0.01) and between TE and CTC (p<0.01). Positive but statistically non-significant relationships were also identified for CN and LEV with SXT, LEV and C, LEV and AMP, CN and AMP, and SMM and SXT (p>0.05, r >0.5). Strongly positive correlations were also reported in other drugs, such as nalidixic acid MICs with cefotaxime and ciprofloxacin MICs [27] and vancomycin with daptomycin resistance in *Staphylococcus aureus* (*S. aureus*) [51]. These correlations indicate that the *E. coli* clones that were tested in this study probably developed co-selection by containing one drug resistance gene on the plasmids that were resistant to other antimicrobials [54]. These correlations also indicate possible similar resistance mechanisms for the different drugs, as reported for *S. aureus,* showing strongly positive correlation between vancomycin and daptomycin susceptibility likely due to the common physical barrier of a thickened cell [51].

**Table 2:** Spearman correlation matrix of the percent resistances to the eight drugs in seven of the nine sampling sites.

	AMP	C	LEV	TE	CN	SXT	SMM	CTC
AMP	1.000	^–^	^–^	^–^	^–^	^–^		
C	0.898^**^	1.000	^–^	^–^	^–^	^–^		
LEV	0.503	0.524	1.000	^–^	^–^	^–^		
TE	0.048	0.286	0.286	1.000	^–^	^–^		
CN	0.587	0.452	−0.048	0.333	1.000	–		
SXT	0.934^**^	0.905^**^	0.548	0.214	0.595	1.000		
SMM	0.311	0.405	0.000	0.071	0.262	0.524	1.000	
CTC	0.036	0.286	0.143	0.952**	0.357	0.119	−0.071	1.000

The sites of Haizi reservoir and Cuo River upstream have been removed based on the low antibiotic levels and few *E. coli* isolates, respectively. AMP = ampicillin, C = chloromycetin, LEV = levofloxacin, TE = tetracycline, CN = gentamycin, SXT = sulfamethoxazole-trimethoprim, SMM = sulfamonomethoxine, CTC = chlortetracycline). ^**^ indicate the significance level of p<0.01.

Table 3 presents the antibiotic resistance rate and profiles of *E. coli* from the sites of the Jiyun River. Although comparatively less *E. coli* were detected in the two sites of Jinji River (Table S2), the isolated cultures exhibited high resistance rates and MAR (Table 3). Spearman correlation analysis revealed no statistical correlations between *E. coli* MPN and resistance rate (r = 0.07, p = 0.85) and between *E. coli* MPN and MAR (r = −0.42, p = 0.27). It suggests the prevalence of antibiotic resistance for *E. coli* in the Jiyun River regardless of the *E. coli* contamination extent. The *E. coli* isolates from each of the three rivers displayed generally consistent increases in both the resistance rates and MAR indexes from up- to midstream. Statistically no-significant difference was found among the three rivers in resistance rates and resistance diversity (MAR indexes) based on a non-parametric k independent sample Kruskal-Wallis test (p = 0.50 for resistance rate and p = 0.10 for MAR). The *E. coli* isolates from different water systems showed varying drug numbers of resistance. The Ju River presented all of the resistance profiles from single- to eight-drug, dominated by one- to five-drug resistance. The Cuo River primarily displayed one-, two- and three-drug resistance. In contrast, the *E. coli* from Jinji River was more chiefly resistant to more than five drugs. In particular, 15% and 22% of *E. coli* isolates in JJ1 and JJ2, respectively, exhibited eight-drug resistance. Similar to the sites prior to intersection, the resistance profiles of *E. coli* at the two intersection sites also covered nearly all of the patterns from one- to seven-drug resistance.

**Table 3:** Overall antibiotic-resistance frequency (%), MAR index and one-, two-, three-, four-, five-, six-drug, seven-drug and eight-drug resistance frequency (%) for *E. coli* isolated from each of the nine sampling sites.

Parameter	Control (n = 38)	J1 (n = 25)	J2 (n = 40)	C1 (n = 2)	C2 (n = 24)	JJ1 (n = 26)	JJ2 (n = 9)	JC (n = 29)	JCJJ (n = 25)
Frequency (%)	82	88	98	50	88	77	100	93	84
MAR index	0.47	0.37	0.40	0.13	0.31	0.46	0.79	0.45	0.29
One-drug (%)	3	23	26	0	10	20	0	7	43
Two-drug (%)	3	27	15	100	29	15	0	22	14
Three-drug (%)	23	5	13	0	52	0	0	19	5
Four-drug (%)	29	18	15	0	5	0	11	11	19
Five-drug (%)	10	9	18	0	0	5	22	19	10
Six-drug (%)	6	5	5	0	0	30	11	15	0
Seven-drug (%)	23	9	8	0	0	15	33	7	10
Eight-drug (%)	3	5	0	0	5	15	22	0	0

Control, Haizi reservoir; J1 and J2, up- and midstream of Ju River; C1 and C2, up- and midstream of Cuo River; JJ1 and JJ2, up- and midstream of Jinji River; JC and JCJJ, intersection sites of Ju River with Cuo River and Jinji River, respectively.

The extent of the antibiotic-resistance of *E. coli* in the Jiyun River maybe is the highest currently reported and was significantly higher than the reports for other agricultural watersheds of British Columbia (Resistance rate: 20–50%) [27], the Beijing Wenyu River (Average rate: 48%, MAR: 0.11–0.14) [26], and the Dongjiang in Guangzhou (MAR: 0.17–0.50) [55]. Furthermore, the extent of resistance in all of these agriculture areas or both agriculture and human-affected areas was greater than the urban surface water in Japan (resistance rate: 37%) and the US (resistance rate: 5–38%) [31], [56]. The generally greater average resistance rate and MAR in surface water of the study agricultural region relative to reported results is probably related to generally higher levels of antibiotic contamination in such areas. In addition to the cause of frequent and long-term exposure to various different levels of antibiotics, special water conditions, such as low flow and high nutrient concentrations, might also contribute to the high antibiotic resistance in the Jiyun Rivers [39], [57], [58].

### Relationships between the resistance frequencies of *E. coli* and the corresponding antibiotics

The correlations between the resistance rate and the detection concentration for the five drugs SMM, CTC, TE, LEV and SXT were separately examined. In general, no clear relationship was observed for any of these drugs (Figure S1). The relationship between the extent of antibiotic resistance or ARG abundance with the antibiotic contents has also been examined in other studies, and contradictory observations have been reported. The abundances of resistance genes are positively related to the antibiotic concentrations in the Haihe and Huangpu Rivers in China [2], [39]. However, no clear correlation was noted between fluoroquinolones and the corresponding resistant *E. coli* in the rivers of Osaka, Japan [3], in agreement with the findings in the present study. The lack of correlation between the antibiotic concentrations and the extent of resistance may be caused by several confounding effects. Antibiotic-resistant bacteria are linked not only to the exposure to corresponding antibiotics but also to other factors, such as the co-selection of toxic heavy metals [59] and the cross-resistance among different types of antibiotics [54], as well as certain water quality variables, such as nutrients, temperature, DO and salinity [27]. In addition to the direct formation of resistance for *E. coli* when exposed to the selective pressure of antibiotics and other factors, *E. coli* can also indirectly obtain resistance through the horizontal ARG transfer among microbial populations [3], [26]. Antibiotics and resistant *E. coli* have different fates and persistence in the environment. For example, antibiotic resistance will remain for at least one month after the disappearance of antibiotics [60]. Considering the complexity of the antibiotic-resistance-formation mechanisms and the influences of various water quality parameters in aquatic environments, more in-depth and systematic studies should be conducted in the future. Regardless, it is clear that a large amount of VAs have been abused in animal production farms and reside in the surrounding aquatic environment, which is likely a primary driver for the emergence of resistant strains in the environment.

The observations in the present study are a cause for concern in terms of the following aspects. First, the Jiyun River is upstream of the Haihe River, which is the largest water system in north China and the main river flowing into Bohai Bay. Thus, high levels of antibiotics and resistant *E. coli* in the Jiyun River serve as potential contamination sources of the Bohai Bay. Second, in most instances, the surface water in rural areas is directly used for agricultural irrigation, which will likely result in the uptake and translocation of antibiotics by plants. After being irrigated with carbamazepine-containing wastewater for 60 and 110 days, carbamazepine was detected in soybean roots and beans [61]. Finally, given that more than 90% of the antibiotic-resistant *E. coli* in aquatic environments is conveyed by corresponding ARGs [26], this resistant *E. coli* might be the potential reservoir of various ARGs, which is particularly worth noting.

### Antibiotics and antibiotic-resistant *E. coli* in the potential drinking water source

Only 12.15 ng L^−1^ antibiotics were detected at the site of the Haizi reservoir, which is the potential drinking water source of the Beijing population. However, 38 *E. coli* isolates were also retrieved from this site that displayed multiple antibiotic resistances (Table 3). The presence of *E. coli* and resistance in the well-protected Haizi reservoir may be caused by input from the upper stretch in Hebei province. Wildlife is also a suspected source because wildlife has been reported as the primary *E. coli* contributor in headwaters and can acquire antibiotic resistance through crossover with livestock animals [62]–[65]. In addition, stationary water flow in such reservoirs is also favorable for resistant bacterial growth and ARG transfer [57], [58]. However, the specific reason is still unclear and requires further examination. The occurrence of antibiotic-resistant microorganisms and ARGs in drinking water sources or headwaters has been reported in other regions, such as the Huangpu River, China [66] and the Elk Creek Watershed, British Columbia [27]. The risk from *E. coli* could be completely eliminated if used for human consumption because the water is required to be treated by the Beijing Water Group Company to reach drinking standards. These results underscore the urgent need to explore the source and risks of resistant bacteria and ARG contamination in drinking water sources.

## Conclusions

This study demonstrated the extensive use and partial abuse of VAs in livestock farming in the study area, which probably is responsible for the prevalence of VAs, particularly hydrophilic VA compounds, in the surrounding surface water. A total of 88% of *E. coli* isolates from these rivers were resistant to one or more antimicrobial agents. Significant correlations were found among the resistance rate of SXT, C and AMP as well as between TE and CTC, suggesting a possible cross-selection for resistance among these drugs. An increasing tendency was observed for total antibiotic contents along the two tributaries, which was most likely attributed to the potential continuous input from animal operations discharge and field runoff. Also, the antibiotics resistance frequency for *E. coli* from midstream is greater than those from upstream for the three rivers. *E. coli* isolates from different water systems showed varying drug numbers of resistance. No obvious correlation was found between the antibiotic resistant rate of *E. coli* and the corresponding antibiotic concentrations, indicating that the resistance formation process must be affected by aquatic factors besides antibiotics. These results provide baseline data on the antibiotics and antibiotic-resistant bacteria contamination that are associated with widespread livestock production. For improved contamination control and environmental protection in rural areas, there is an urgent need to develop management protocols in the animal industry. In addition, the occurrence of resistance genes and potential threats to agricultural ecosystem safety should also be noted, as water is the main interface of antibiotics, resistant bacteria and ARG transfer in the ecosystem.

## Supporting Information

Figure S1 Relationships between CTC resistance rate versus CTC concentration (a), TE resistance rate versus TC concentration (b), SMM resistance rate versus SMM concentration (c), SXT resistance rate versus SMX concentration (d) and LEV resistance rate versus OFC concentration (e) in seven of the nine sampling sites. The sites of Haizi reservoir and Cuo River upstream have been removed based on the low antibiotic levels and few *E. coli* isolates, respectively. TE, tetracycline; CTC, chlortetracycline; SXT, sulfamethoxazole-trimethoprim; SMM, sulfamonomethoxine; LEV, levofloxacin; TC, tetracycline; CTC, chlortetracycline; OFC, ofloxacin; SMX, sulfamethoxazole; SMM, sulfamonomethoxine.
(TIF)

Table S1 GPS coordinates (deg./min./sec.) of the 12 water sample sites in study region (Control, Haizi reservoir; J1, J2 and J3, up-, mid- and downstream of Ju River; C1 C2, and C3, up-, mid- and downstream of Cuo River; JJ1, JJ2, and JJ3, up-, mid- and downstream of Jinji River; JC and JCJJ, intersection sites of Ju River with Cuo River and Jinji River, respectively).
(DOCX)

Table S2 Most possible number (MPN) of the *E. coli* in the Jiyun River (MPN/100 ml) (Control, Haizi reservoir; J1, and J2, up- and midstream of Ju River; C1 and C2, up- and midstream of Cuo River; JJ1 and JJ2, up- and midstream of Jinji River; JC and JCJJ, intersection sites of Ju River with Cuo River and Jinji River, respectively.).
(DOCX)

Table S3 Detection frequencies, ranges and means of the 12 target antibiotics in sediment of the Jiyun River.
(DOCX)
