# Supplementary material for: Prevalence of Veterinary Antibiotics and Antibiotic-Resistant Escherichia coli in the Surface Water of a Livestock Production Region in Northern China
Source: PLoS One. 2014 Nov 5;9(11):e111026. doi: 10.1371/journal.pone.0111026 (PMC4220964; doi:10.1371/journal.pone.0111026)
Supplement: Table S1 — GPS coordinates (deg./min./sec.) of the 12 water sample sites in study region (Control, Haizi reservoir; J1, J2 and J3, up-, mid- and downstream of Ju River; C1 C2, and C3, up-, mid- and downstream of Cuo River; JJ1, JJ2, and JJ3, up-, mid- and downstream of Jinji River; JC and JCJJ, intersection sites of Ju River with Cuo River and Jinji River, respectively). (DOCX) [file pone.0111026.s002.docx]

**Table S1** GPS coordinates (deg./min./sec.) of the 12 water sample sites in study region (Control, Haizi reservoir; J1, J2 and J3, up-, mid- and downstream of Ju River; C1 C2, and C3, up-, mid- and downstream of Cuo River; JJ1, JJ2, and JJ3, up-, mid- and downstream of Jinji River; JC and JCJJ, intersection sites of Ju River with Cuo River and Jinji River, respectively).

| Site | Longitude | Latitude |
| --- | --- | --- |
| Control | 117.18.18 | 40.11.12 |
| J1 | 117.07.14 | 40.07.39 |
| J2 | 117.05.26 | 40.07.39 |
| J3 | 117.04.05 | 40.06.28 |
| C1 | 117.02.35 | 40.10.30 |
| C2 | 117.04.27 | 40.09.36 |
| C3 | 117.04.05 | 40.08.07 |
| JJ1 | 116.52.10 | 40.10.46 |
| JJ2 | 116.54.02 | 40.09.10 |
| JJ3 | 116.55.45 | 40.06.42 |
| JC | 117.02.43 | 40.06.28 |
| JCJJ | 117.00.37 | 40.04.23 |
