# Supplementary material for: Prevalence of Veterinary Antibiotics and Antibiotic-Resistant Escherichia coli in the Surface Water of a Livestock Production Region in Northern China
Source: PLoS One. 2014 Nov 5;9(11):e111026. doi: 10.1371/journal.pone.0111026 (PMC4220964; doi:10.1371/journal.pone.0111026)
Supplement: Table S2 — Most possible number (MPN) of the E. coli in the Jiyun River (MPN/100 ml) (Control, Haizi reservoir; J1, and J2, up- and midstream of Ju River; C1 and C2, up- and midstream of Cuo River; JJ1 and JJ2, up- and midstream of Jinji River; JC and JCJJ, intersection sites of Ju River with Cuo River and Jinji River, respectively.). (DOCX) [file pone.0111026.s003.docx]

**Table S2** Most possible number (MPN) of the *E. coli* in the Jiyun River (MPN/100 ml) (Control, Haizi reservoir; J1, and J2, up- and midstream of Ju River; C1 and C2, up- and midstream of Cuo River; JJ1 and JJ2, up- and midstream of Jinji River; JC and JCJJ, intersection sites of Ju River with Cuo River and Jinji River, respectively.).

| Control | J1 | J2 | C1 | C2 | JJ1 | JJ2 | JC | JCJJ |
| --- | --- | --- | --- | --- | --- | --- | --- | --- |
| 74.4 | 82 | 125.6 | 60.4 | 47.1 | 17.6 | 8.2 | 86.3 | 3419 |
