# Supplementary material for: Prevalence of Veterinary Antibiotics and Antibiotic-Resistant Escherichia coli in the Surface Water of a Livestock Production Region in Northern China
Source: PLoS One. 2014 Nov 5;9(11):e111026. doi: 10.1371/journal.pone.0111026 (PMC4220964; doi:10.1371/journal.pone.0111026)
Supplement: Table S3 — Detection frequencies, ranges and means of the 12 target antibiotics in sediment of the Jiyun River. (DOCX) [file pone.0111026.s004.docx]

**Table S3** Detection frequencies, ranges and means of the 12 target antibiotics in sediment of the Jiyun River.

| Antibiotic |  | Frequency (%)  （）（）（）  （%） | | | | Range (ng g^-1^) | | Mean (ng g^-1^) | |
| --- | --- | --- | --- | --- | --- | --- | --- | --- | --- |
| Tetraycline (TC) | | |  | 66.67 | n.d-15.70 | | 5.16 | |  |
| Oxytetracycline (OTC) | | |  | 91.67 | n.d-121.00 | | 35.36 | |  |
| Chlortetracycline (CTC) | | |  | 100.00 | 2.70-237.00 | | 74.84 | |  |
| Doxycycline (DOC) | | |  | 66.67 | n.d-16.20 | | 5.22 | |  |
| Ciprofloxacin (CFC) | | |  | 100.00 | 2.36-3.81 | | 2.68 | |  |
| Enrofloxacin (EFC) | | |  | 25.00 | n.d-0.27 | | 0.06 | |  |
| Ofloxacin (OFC) | | |  | 100.00 | 0.62-8.77 | | 1.64 | |  |
| Sulfadiazine (SDZ) | | |  | 58.00 | n.d-3. 26 | | 0.53 | |  |
| Sulfamethoxazole (SMX) | | |  | 100.00 | 0.95-5.81 | | 1.60 | |  |
| Sulfamonomethoxin (SMM) | | |  | 25.00 | n.d-3.19 | | 0.53 | |  |
| Sulfameter (SM) | | |  | 83.33 | n.d-6.79 | | 1.75 | |  |
| Sulfachinoxalin (SCX) | | |  | 100.00 | 0.07-0.55 | | 0.21 | |  |

n.d: non-detected
